# Calibration of the PA6 Short-Fiber Reinforced Material Model for 10% to 30% Carbon Mass Fraction Mechanical Characteristic Prediction

**DOI:** 10.3390/polym14091781

**Published:** 2022-04-27

**Authors:** Evgenii Kurkin, Mariia Spirina, Oscar Ulises Espinosa Barcenas, Ekaterina Kurkina

**Affiliations:** Joint Russian-Slovenian Laboratory Composite Materials and Structures, Samara National Research University, 34 Moskovskoe Shosse, Samara 443086, Russia; marie.spirina@yandex.ru (M.S.); oscar.espinosa.barcenas@gmail.com (O.U.E.B.); ekaterina.kurkina@mail.ru (E.K.)

**Keywords:** composite, mechanical characteristics, material model, short fibers, polyamide 6, fiber mass fraction

## Abstract

Short-fiber reinforced composites are widely used for the mass production of high-resistance products with complex shapes. Efficient structural design requires consideration of plasticity and anisotropy. This paper presents a method for the calibration of a general material model for stress–strain curve prediction for short-fiber reinforced composites with different fiber mass fractions. A Mori–Tanaka homogenization scheme and the J2 plasticity model with layered defined fiber orientation were used. The hardening laws: power, exponential, and exponential and linear were compared. The models were calibrated using experimental results for melt front, orientation tensor analysis, fiber length, and diameter and tension according to ISO 527-2, for samples of PA6 which were either non-reinforced, or reinforced with 10%, 15%, 20%, and 30% carbon fiber mass fractions. The novelty of this study lies in the transition from the strain–stress space to the strain–stress–fiber fraction space in the approximation of the material model parameters. We found it necessary to significantly reduce the fiber aspect ratio for the correct prediction of the mechanical characteristics of a composite using the Mori–Tanaka scheme. This deviation was caused by the ideal solution of ellipsoidal inclusion in this homogenization scheme. The calculated strength limits using Tsai–Hill failure criteria, based on strain, could be used as a first approximation for failure prediction.

## 1. Introduction

Reinforced polyamide 6 (PA6), like many other reinforced thermoplastics, has found applications in durable goods, computer hardware, biomedical, automobile, and aerospace sectors [1,2,3,4]. For example, in the aerospace industry, short-fiber reinforced thermoplastics have been utilized in several components of A340-600 and C295 [5], A350 XWB [6], and V-22 tilt-rotor aircraft [7]. Reinforced thermoplastics are of interest for their high performance in structural applications, low-cost manufacturing process, ease of manipulation [1], and ability to meet waste and recycling regulations [8,9,10]. However, the difficulty of predicting the anisotropy throughout the design process leads to their implementation in secondary components more than in primary structures [11,12]. For example, accounting for the effect of anisotropy on the mechanical behavior of the components [13,14,15,16,17,18] requires knowledge of both the fiber length distribution and the fiber orientation distribution, which depend on the fiber content, the geometry of the mold, the processing conditions, and the injection gate [19,20].

Currently, anisotropy is considered in structural analysis by implementing a solution to the inclusion problem by introducing Eshelby’s tensor [21], and a micromechanical method such as the Mori–Tanaka model for predicting the effective properties of two-phase composites [22]. Contemporary investigation has focused on the mechanical behavior of short-fiber reinforced polymers (SFRP) with high fiber volume fractions [15,16], and the theory presented by Fu. S.-Y. on modeling SFRP [13,14] provides evidence of the importance of studying the influence of the fiber content on the mechanical characteristics of the polymers [23]. Furthermore, creating a more precise short-fiber model is a relevant topic that has been addressed by [24,25]. Although these studies increase the reliability of modeling for anisotropy, data for SFRP are generally provided for a specific fiber volume fraction, which limits the material model. Hence, selecting the material in the early stages of design requires the usage of an accurate material model with the ability to adjust the fiber weight fraction.

This study presents a general material model for PA6 reinforced with different fiber mass fractions. To create the model, experimental studies were conducted using PA6 reinforced with different short carbon fiber weight fractions. The results of the experiments were used to obtain the mechanical characteristics as well as the fiber length and fiber orientation distributions, which were subsequently used for modeling the material using a second-order Mori–Tanaka homogenization scheme and three different hardening laws. The material models obtained are presented alongside their calibration data, which were obtained by applying curve fitting through optimization of the material parameters.

## 2. Materials and Methods

The creation of a composite material model requires knowledge of the mechanical characteristics of its components. We started by obtaining the mechanical characteristics of non-reinforced PA6 using a tensile test. The fiber aspect ratio was then determined by burning a sample of reinforced PA6. The unidirectional short-fiber reinforced composite was modeled as a transversely isotropic material by application of the Mori–Tanaka homogenization scheme. Tucker’s procedure was applied to transform the material from a unidirectional transversely isotropic one into a composite, which depended on the fiber orientation. We assumed that the stress and strain of the composite depend on its percentage content fractions. The plasticity of the matrix was modeled using the J2 plasticity model. The following three different laws were used to model hardening: the power, the exponential, and the exponential and linear laws.

### 2.1. Plate Molding Experiment

**Figure 1:**
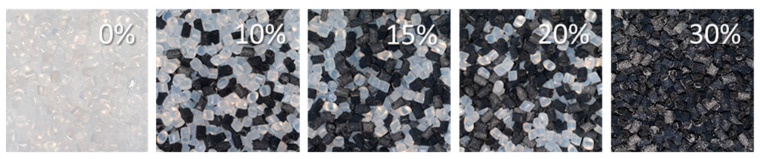
Mixed pellets to obtain different fiber mass fraction.

The filling process dictates the fiber orientation. The effect of the fiber mass fraction on the filling process and mechanical characteristics of short-fiber PA6 with different fiber mass fractions was investigated. The weight of each pellet was measured using an electronic balance (0.01 g resolution), and its size was measured using a Vernier caliper (0.1 mm resolution). PA6, and PA6 reinforced with carbon fiber, had a mass of 0.0102 g and 0.0048 g, respectively; a length of 3.3 ± 0.17 and 2.02 ± 0.11 mm, respectively; and a diameter of 2.3 ± 0.09 and 2.27 ± 0.14 mm, respectively. Figure 1 shows the pellets used for experimental investigation: non-reinforced PA6 (matrix material), short-fiber reinforced PA6 with 30% carbon fiber mass fraction (Gamma Plast UPA6—30 M), and a combination of both materials mixed in mass proportions of 1:2, 1:1, and 2:1 to fabricate the fiber mass fractions of 10%, 15%, and 20%, respectively. A standard cement mixer was used for mixing all materials until the mixture was homogeneous. The pellets were dried at a temperature of 90 C for 4 h in a plastic pellet dryer before injection.

**Figure 2:**
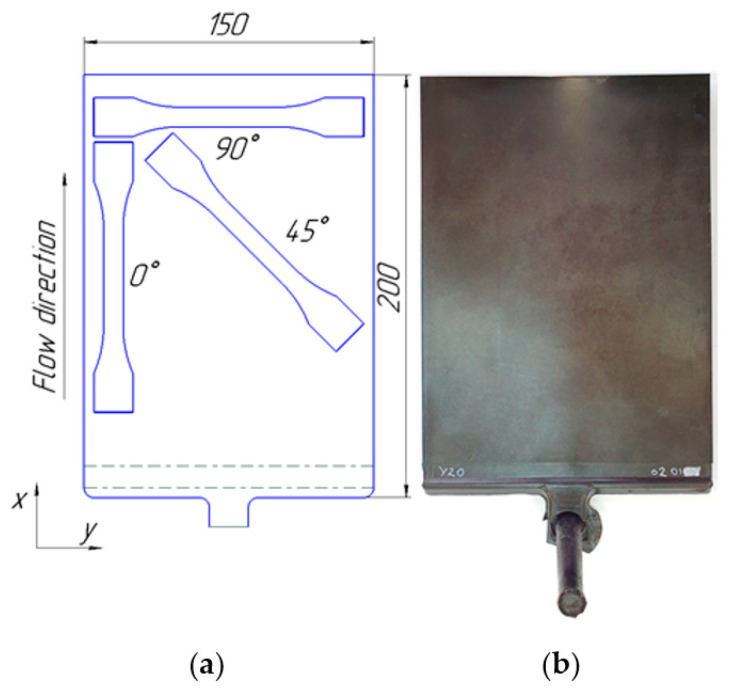
Plate molding: (**a**) Size and ISO 527-2 specimen layout; (**b**) Example manufactured plate.

Tensile tests using 1B samples as specified by the ISO 527-2 standard were performed to obtain the mechanical characteristics of the material. The samples were cut from plates with the dimensions 200 mm × 150 mm × 4 mm (Figure 2a). The manufactured plates are shown in Figure 2b. Plates of different fiber compositions were injected using a Negri Bossi VE 210-1700 injection molding machine. The filling parameters were as follows: melt temperature 225 °C, mold temperature 80 °C, and flow rate 42 cm^3^/s.

### 2.2. Investigation of Fibers under Scanning Electronic Microscope

**Figure 3:**
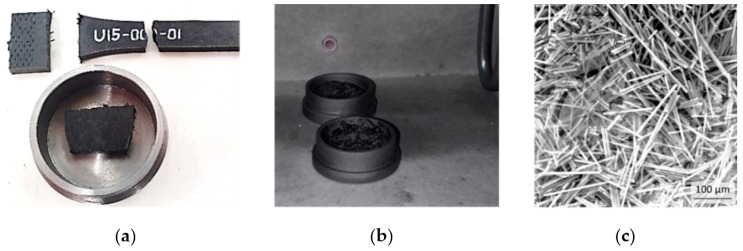
Short fiber extraction from composite: (**a**) Sample preparation; (**b**) burning samples in the oven; (**c**) fibers after matrix burning.

Modeling the mechanical behavior of the short-fiber reinforced PA6 requires knowledge of the aspect ratio (AR) between the diameter and the length of the fibers [26,27,28]. Measuring the dimensions of the fibers can be achieved if the polymer containing the fibers is degraded at an elevated temperature (Figure 3). The technique used by [29] was taken as a basis for the fiber acquisition. Samples with fiber mass fractions of 15% (Figure 3a) and 30% were cut from the molded plates and burnt in an inert atmosphere oven (Figure 3b). The oven’s camera was filled with a nitrogen atmosphere to avoid fiber degradation, and maintained without a flow rate. The process was initiated at 20 °C with a heating rate of 5 °C/min and reached a temperature of 900 °C that was held for 20 min, before cooling at a rate of −5 °C/min. The fibers obtained were investigated under a Tescan Vega electronic microscope (Figure 3c).

### 2.3. The Effective Fiber Length

For correct modeling and prediction of the fiber strength, the concept of the effective fiber length was introduced. It was first considered by Rosen [30] in 1965 while describing the mechanical characteristics of continuously reinforced composites. In [31], the effective fiber length is known as the debonding fraction due to the substitution of the ellipsoidal inclusion for the “equivalent debonded inclusion”, and in that study the fraction of the debonded interface surface was 0.26. In the present work, the effective fiber length was estimated quantitatively. This technique may be described using the following formula:

(1) $$\varphi =\frac{AR_{model}}{AR_{experimental}}$$

### 2.4. Calculation of Short-Reinforced Composite Tensile Curves

The material modelling was performed in Digimat-MF, while the parameters were identified using Digimat-MX RVE. The microstructure consisted of two phases—the elastoplastic PA6 matrix, and the elastic short-carbon fibers modeled as elliptic inclusions. The models of the matrix were obtained after performing curve fitting of the tensile test results for the specimens without reinforcement.

The fibers (inclusions) and the matrix were homogenized via Digimat-MF, using a second-order Mori–Tanaka homogenization scheme for the computation of the mechanical properties. The unidirectional short-fiber reinforced composite was modeled as a transversely isotropic material. The elastic moduli introduced by Tandon and Weng [32] were used to calculate the elastic coefficients:

(2) $$\frac{E_{11}}{E_{m}}=\frac{1}{1+\frac{\phi_{f}\left(A_{1}+2\upsilon_{m}A_{2}\right)}{A_{6}}}$$

(3) $$\frac{E_{22}}{E_{m}}=\frac{1}{1+\frac{\phi_{f}\left[-2\upsilon_{m}A_{3}+\left(1-\upsilon_{m}\right)A_{4}+\left(1+\upsilon_{m}\right)A_{5}A_{6}\right]}{2A_{6}}},$$

where $E_{m}$ and $\upsilon_{m}$ are the Young’s modulus and Poisson ratio of the matrix, respectively. The volume fraction of the fiber is represented by $\phi_{f}$. The parameters $A_{i}$ are the functions of Eshelby’s tensor and can be found in [33]. In this study, we used Eshelby’s tensor for elliptical inclusion, and this depends on the fiber aspect ratio. The Tucker’s averaging procedure was used to determine the fiber orientation tensor, which is described as follows:

(4) $$\begin{array}{cc} C_{ijkl} & =B_{1}a_{ijkl}+B_{2}\left(a_{ij}\delta_{kl}+\delta_{ij}a_{kl}\right)+B_{3}\left(a_{ik}\delta_{jl}+a_{il}\delta_{jk}+a_{jl}\delta_{ik}+a_{jk}\delta_{il}\right)\\ & +B_{4}\left(\delta_{ij}\delta_{kl}\right)+B_{5}\left(\delta_{ik}\delta_{jl}+\delta_{il}\delta_{jl}\right), \end{array}$$

where *a_ijkl_* is the fourth-order fiber orientation tensor, δ*_ij_* is the second-order unit tensor, and the coefficients *B* are related to the components of the stiffness matrix of the transversely isotropic unidirectional composite [34]. The fourth-order tensor in Tucker’s averaging procedure was reduced to a second-order tensor by applying the orthotropic closure approximation presented by Cintra and Tucker in [35]. The approximation parameters of the fiber orientation tensors mainly influence the calculation of the stress–strain curves. The fiber direction from 0 to 90° was divided into 20 equal parts, with a tolerance interval in the fiber orientation tensor of 0.01.

The composite stress and strain depend on the stress and strain of the matrix and fiber, proportional to their volume fractions:

(5) $$\varepsilon =\left(1-\phi_{f}\right)\varepsilon_{m}+\phi_{f}\varepsilon_{f},$$

(6) $$\sigma =\left(1-\phi_{f}\right)\sigma_{m}+\phi_{f}\sigma_{f}.$$

The stress–strain state of the matrix is described using the J2 plasticity model [36], based on the von Mises equivalent stress, $\sigma_{eq}.$ When $\sigma_{eq}$ exceeds the initial yield stress, the response becomes nonlinear and plastic deformation appears. Plastic strength is expressed as follows:

(7) $$\sigma_{eq}=\sigma_{Y}+R\left(\varepsilon_{p}\right),$$

where $\sigma_{Y}$ is the initial yield stress; $R\left(\varepsilon_{p}\right)$ is the isotropic strain hardening function; and $\varepsilon_{p}$ is the accumulated plastic strain.

Poisson’s ratio of the matrix, in the plastic range, is predicted through Lame parameters using spectral decomposition [37]; elastic bulk module K is taken as a constant, and shear moduli *G* will be

(8) $$G=G_{e}\left(1-\frac{3G_{e}}{3G_{e}+\frac{d\;R\left(\varepsilon_{p}\right)}{d\;\varepsilon_{p}}}\right),$$

where *G_e_*, the elastic shear modulus, is defined using a Lame parameter, based on the given Young’s modulus and the Poisson’s ratio of the matrix in the elastic range.

For all models so described, the matrix Poisson’s ratio in the elastic range is equal to the experimentally measured average value. Poisson’s ratio of the matrix in the elastic range had a slight effect on the stress–strain curves for reinforced PA6 when reverse-engineering the curves, and was not accurate, therefore, Poisson’s ratio of the matrix in the elastic range was excluded from the reverse-engineered parameters.

A comparison between the three hardening stress functions is provided in [38]:

- Power law [39]:
  (9) $$R\left(\varepsilon_{p}\right)=k\varepsilon_{p}{}^{m};$$
- Exponential law:
  (10) $$R\left(\varepsilon_{p}\right)=R_{\infty }\left[1-e^{-m\varepsilon_{p}}\right];$$
- Exponential and linear law:
  (11) $$R\left(\varepsilon_{p}\right)=k\varepsilon_{p}+R_{\infty }\left[1-e^{-m\varepsilon_{p}}\right].$$
  where k is linear hardening modulus, MPa; *m* is hardening exponent; and $R_{\infty }$ is hardening modulus, MPa.

## 3. Results

### 3.1. Melt Front and Microstucture Experimental Investigation

**Figure 4:**
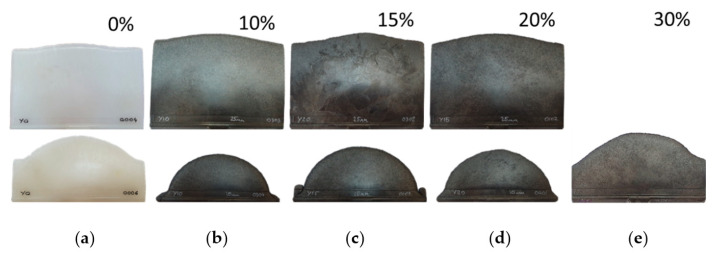
Experimentally obtained plate melt front at molding composites with different fiber mass fraction: (**a**) 0%; (**b**) 10%; (**c**) 15%; (**d**) 20%; (**e**) 30%.

An experimental study of the melt front was conducted by studying partially filled molds (Figure 4). This study allowed us to verify the plastic injection molding models and showed that the fiber mass fraction had a small effect on the melt front of the plates.

**Figure 5:**
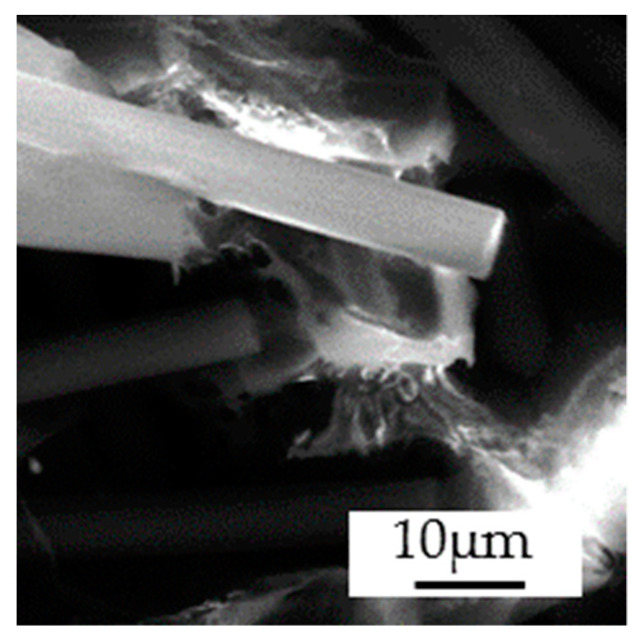
Morphology of the fracture of short-fiber reinforced PA6 with 30% carbon-fiber mass fraction.

The fiber orientation determines the mechanical characteristics of the material to a great extent. To investigate the fiber orientation at the resultant fracture location, a sample was extracted from the 90° tensile test specimens with fiber mass fractions of 15% and 30% (Figure 2a). The shape and roughness of the fibers have a significant influence on the adhesion quality and strength of the composite material [40,41]. The morphology of the fracture surface is shown in Figure 5, it can be observed in the pulled-out fiber and defects in the background.

**Figure 6:**
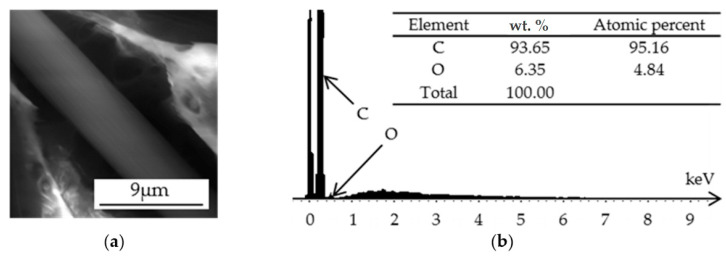
Reinforced PA6 with 30 wt. % short-carbon fiber: (**a**) SEM; (**b**) XRF spectra.

Several factors characterize composite failure, including the adhesion between the matrix and the fiber. The failure surface of the 90° sample with 30 wt. % was analyzed by X-ray fluorescence under a scanning electron microscope (Figure 6). The spectrum on the center of the fiber surface (Figure 6a) consisted of 93.65% C and 6.35% O (Figure 6b), showing a low presence of PA6.

**Figure 7:**
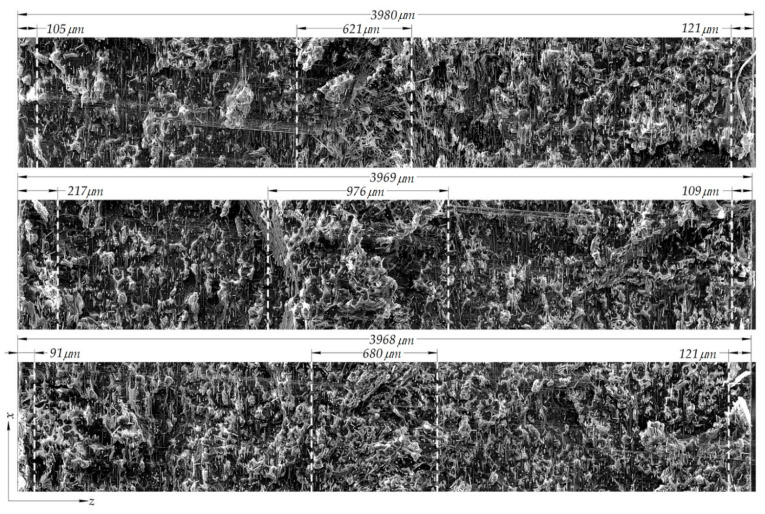
Fracture surface of short-fiber reinforced PA6 with 15% carbon-fiber mass fraction, ISO 527-2 sample, orientated at 90°. Dashed lines demark the size in micrometers of the skin and core layer.

The sample was examined in three places at the failure line; the images obtained under the microscope are shown in Figure 7. Qualitative evaluation of the fiber orientation tensor was performed by comparing the size of the layer against the fiber orientation tensor components. The average values of the specific skin layer, the core layer thickness, and the thickness of the samples with 15% fiber mass fraction, were 7.31%, 25.79%, and 3.98 mm, respectively. In Figure 7, in the shell layer (the layer between the skin and the core layers), the fibers are oriented along the x-axis, which corresponds to the fiber orientation tensor values of the component a_11_. In addition, the fiber orientation tensor predicts that the component a_11_ has the highest probability. The fibers presented a chaotic appearance within the core layer. The skin layer comprised approximately 3% of the thickness, while the core layers constituted 19%. The start and end of every layer were difficult to determine, especially at the transition from the skin layer to the shell layer.

### 3.2. Injection Molding and the Fiber Orientation Models Validation

**Figure 8:**
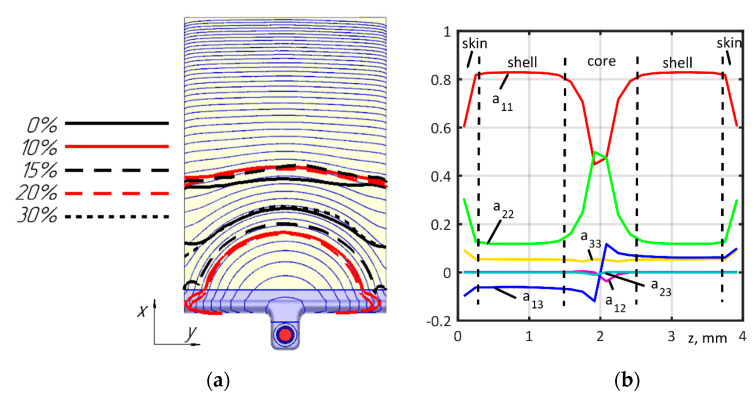
Comparison of simulated and experimental filling: (**a**) Melt front, blue lines—calculated isolines at each 2% of filling time, black and red lines—experimentally obtained melt fronts; (**b**) Orientation tensor components, solid lines—calculated values, dash lines—mean experimentally obtained zones sizes.

The injection molding simulation of PA6 reinforced with 30% mass fiber fraction was performed using Moldex3D R17 on a structured mesh of 4,499,918 elements. The number of elements in the plate (without runner and sprue) along the x-axis was 600, along the y-axis 296, and along the z-axis 24; this totaled 4,262,400 elements. A comparison of the simulated and experimental filling processes is shown in Figure 8a. The fiber orientation tensor is shown in Figure 8b and Table 1, and used in the models of the mechanical characteristics of the materials.

**Figure 9:**
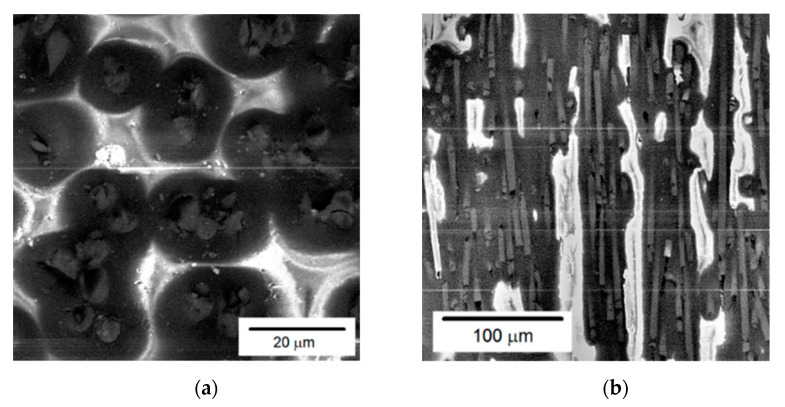
The microstructure of short-fiber reinforced PA6 with 30% carbon-fiber mass fraction along the following orientations: (**a**) 0°; (**b**) 90°.

Quantitative evaluation was performed by comparing the components of the fiber orientation tensor (Figure 8b) against the experimental data presented by Foss et al. [42], and we obtained a reasonable agreement. Microstructure analysis at the orientations 0 and 90° are shown in Figure 9.

### 3.3. Determination Length and Diameter of the Fibers

**Figure 10:**
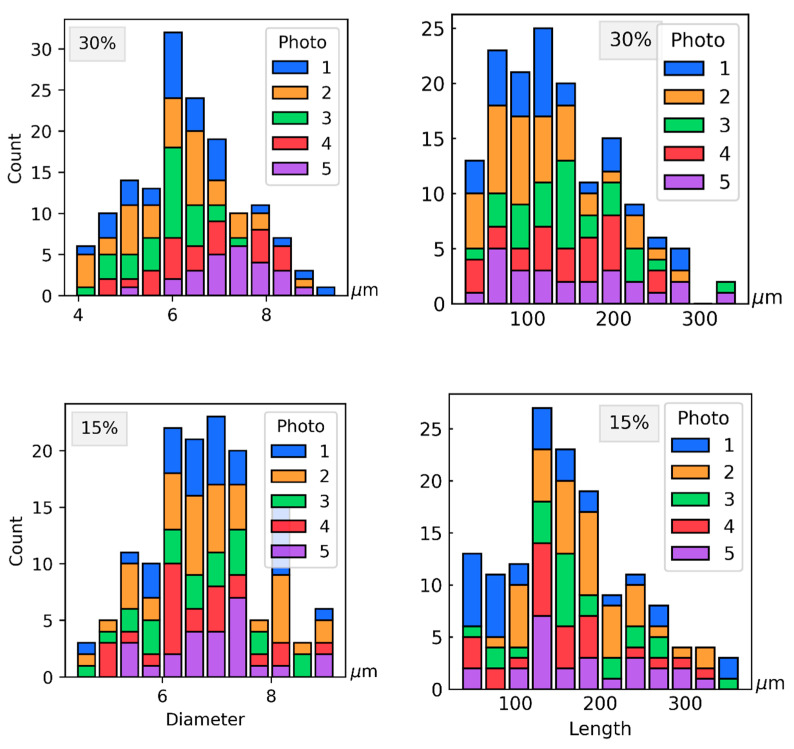
Distribution of diameter and length in 30% and 15% fiber mass fraction samples.

**Figure 11:**
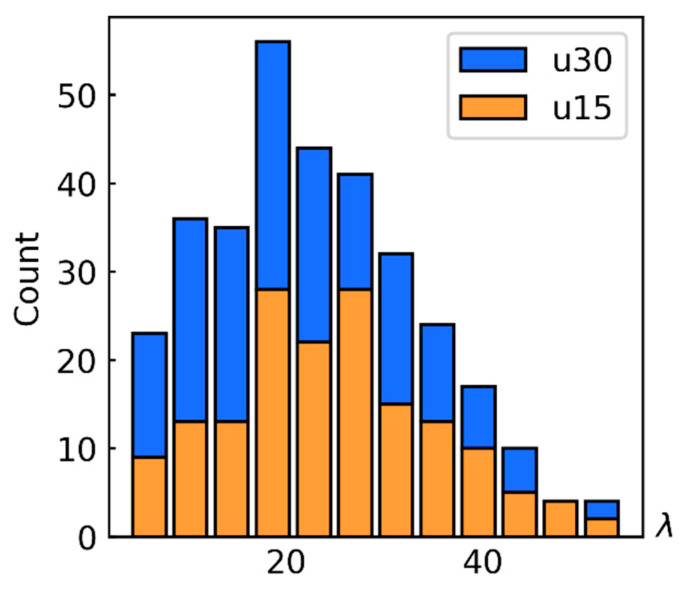
Measured fiber aspect ratio distribution for 15% (u15) and 30% (u30) fiber mass fractions.

To measure the size of the fibers, five images were recorded for each material using a scanning electronic microscope. From every image, 25 to 35 different fibers were measured. In the sample with a 15% fiber mass fraction, it was found that the diameter had a mean of 6.8 μm and a standard deviation of 1.04 μm, and the length had a mean of 167.41 μm and a standard deviation of 72.22 μm. In the sample with a 30% fiber mass fraction, was found that the diameter had a mean of 6.33 μm and a standard deviation of 1.14 μm, and the length had a mean of 135.66 μm and a standard deviation of 67.06 μm. The distribution of the diameter and length of the fibers is presented in the form of histograms in Figure 10. The AR of the fibres was calculated by dividing their measured lengths by their mean diameter (equal to 6.80 for the 15%, and 6.33 for the 30% fiber mass fractions). The AR distribution is presented in Figure 11.

### 3.4. Experimental Stress–strain Curves

Tensile tests were performed for construction of the material models according to ISO 527-2 on 1B specimens from non-reinforced PA6 and composites with 10%, 15%, 20%, and 30%. For each material, nine samples were investigated, cut out from the plates at 0° (red lines), 45° (green lines), and 90° (blue lines) to the flow direction (Figure 2a). The test machine MTS 322.21, as well as the biaxial extensometer MTS 632.85F-14, were used for registering the force and displacement of the specimens.

**Figure 12:**
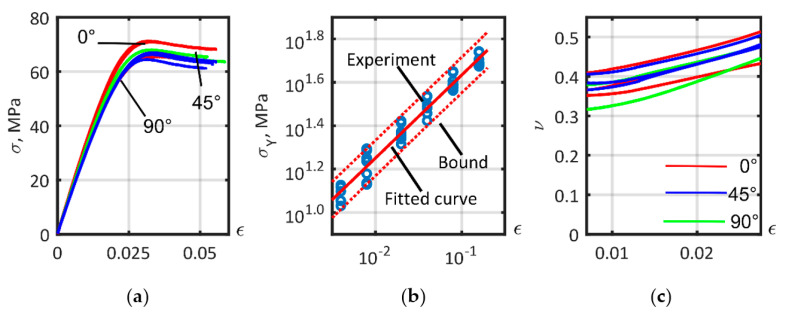
Tensile test of ISO 527-2 PA6 matrix samples: (**a**) Tension curves; (**b**) Yield stress; (**c**) Poisson’s ratio.

PA6 stress–strain curves are shown in Figure 12a; due to its isotropic composition a low variation in mechanical behavior existed between specimens (red lines—0°, green lines—45°, blue lines—90° specimens). The plastic behavior of the material is unreliable in determining the value of the elastic behavior limit. The offset yield point, $\sigma_{Y},$ depends on the offset plastic strain, $\varepsilon_{p}$, and is shown in Figure 12b with a logarithmic scale. The dependence $\sigma_{Y}\left(\varepsilon_{p}\right)$ can be approximated as $lg\left(\sigma_{Y}\right)=p_{1}lg\left(\varepsilon_{p}\right)+p_{2}$, where the coefficients (with a 95% confidence interval) are $p_{1}=0.383\left(0.3615,\;0.4044\right)$ and $p_{2}=1.978\left(1.945,\;2.012\right)$. Poisson’s ratio is shown in Figure 12c. The values for Poisson’s ratio were calculated in a small strain region, and ranged from 0.31 to 0.42, with a mean of 0.3721. The Poisson’s ratio obtained satisfactorily agrees with that presented by D.V. Rosato [43]. The Poisson’s ratio value of 0.3721 was used for the matrix of all material models.

**Figure 13:**
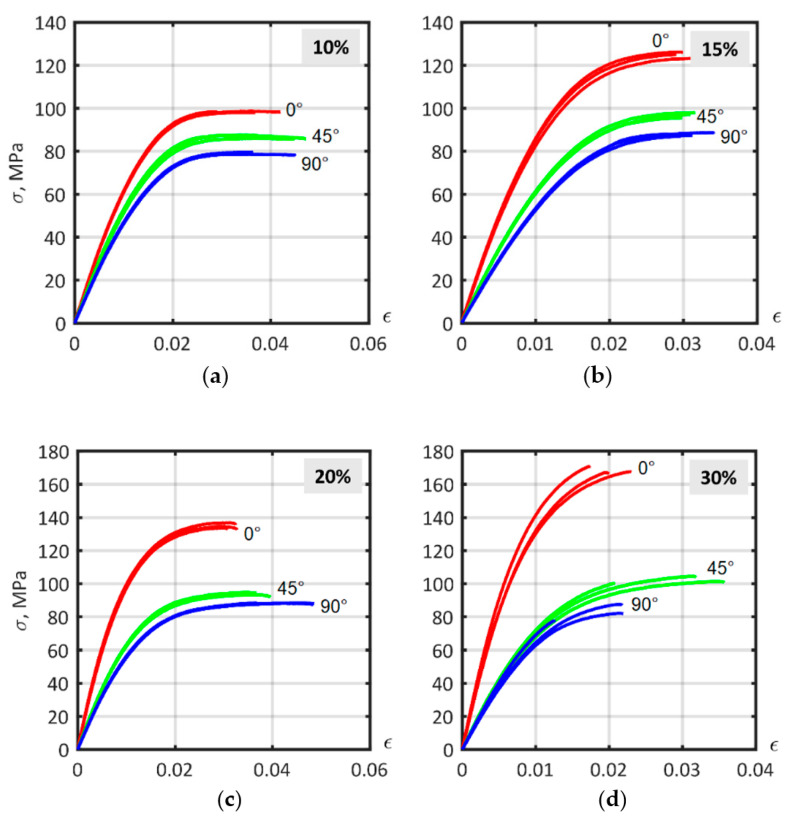
Experimental stress–strain curves for ISO 527-2 samples of PA6 composites with different fiber mass fractions, cut at 0°, 45° and 90° angles to flow direction: (**a**) 10%; (**b**) 15%; (**c**) 20%; (**d**) 30%.

Stress–strain curves of short-fiber reinforced PA6 with different carbon-fiber mass fractions are shown in Figure 13. As expected, samples with a lower fiber content showed behavior closer to that of an isotropic material. Increasing the fiber mass fraction content increased the anisotropic behavior of the material. Moreover, the strength of the 0° samples increased more than the strength of 45° and 90° samples. Specimens, especially at 0° with higher fiber content, were more fragile—the value of ultimate strain decreased. The stress–strain curves of the samples with a 30% fiber mass fraction show the differences between them.

### 3.5. Determination of the Matrix Material Models

**Figure 14:**
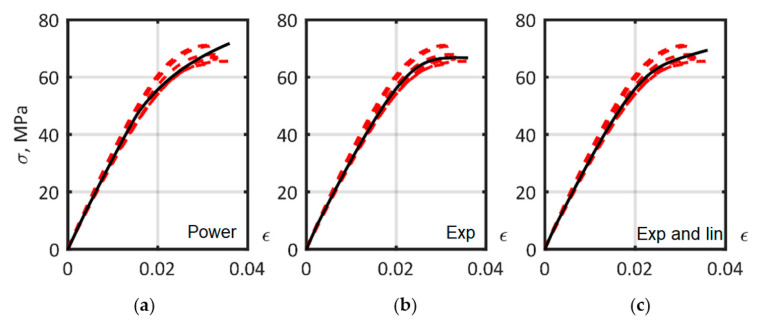
The approximation of PA6 matrix ISO 527-2 tension curves, dashed lines—experiment, solid lines—model, with hardening laws: (**a**) power; (**b**) exponential; (**c**) exponential and linear. The solid lines show the optimized curves. Dashed lines are the experimental data.

Results of the ISO 527-2 tensile test for the PA6 matrix are presented in Figure 14 with dashed lines. The scatter of experimental stress measurements when strain equaled 0.03 had a standard deviation of 2.49 MPa, and a coefficient of variation of 3.71%. The identification of the parameters of the matrix models, including the parameters of the hardening laws in Equations (9)–(11), was established using the Digimat-MX RVE curve fitting module, and was based on the tensile tests presented in Figure 12a. To estimate the accuracy of the approximation, the averaged relative error for each hardening law was obtained [38]. The parameters of the matrix models and the relative errors of the approximations are provided in Table 2. Approximated curves are presented in Figure 14.

### 3.6. Comparison of the Effect of Considering the Distribution of Fiber Lengths and the Distribution of the Orientation Tensor on the Accuracy of Approximation of the Tensile Curves of a Short-Reinforced Composite

**Figure 15:**
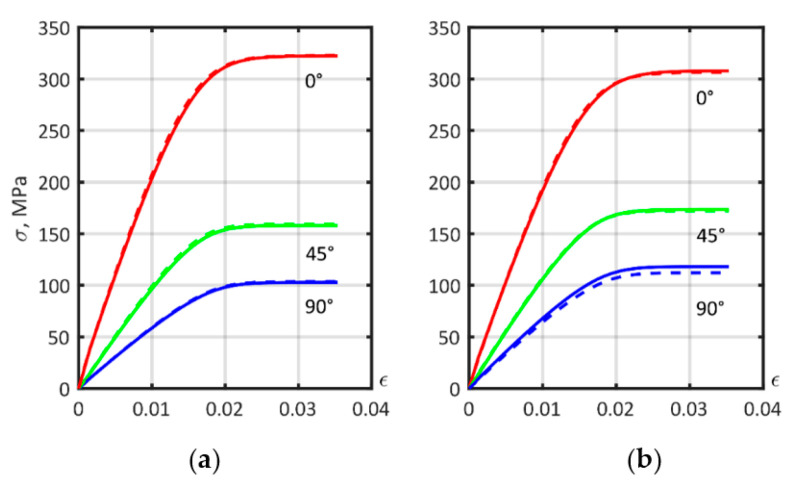
Stress–strain curves of short-fiber reinforced PA6 with 30% carbon fiber were modeled using the exponential law. Comparison between: (**a**) Single AR value and AR distribution; (**b**) Single layer and multilayer analysis.

During model construction, it was impossible to simultaneously consider the distribution of the AR of the fibers and the distribution of the fiber orientation tensor over the thickness of the sample. The stress–strain curves of short-fiber reinforced PA6 with a 30% carbon-fiber mass fraction, presented in Figure 15, were created using the exponential law. In Figure 15a, a comparison between single layer and multilayer (using Table 1) orientation tensor definitions, with a single AR value of 26.5 is presented. Figure 15b compares a fixed AR of 26.5 with the AR distribution according to Figure 11. It can be concluded that allowing for the layered orientation tensor had a more noticeable effect on the simulation of a 90° sample, whereas accounting for AR distribution could be replaced by an equivalent constant value.

### 3.7. Determination of the Parameters of the Composite Material Models, Common for Different Fiber Mass

**Figure 16:**
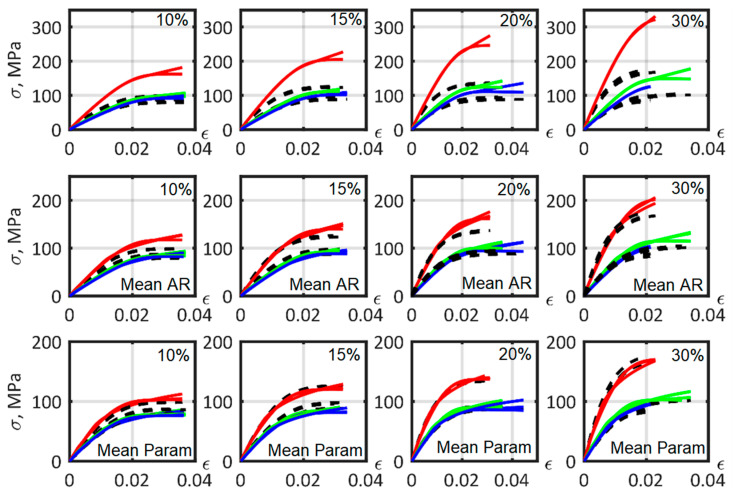
Stress–strain curves for the model material for PA6 with different short carbon fiber mass fractions, orientated in different directions. From left to right: 10%, 15%, 20%, and 30% fiber mass fraction. From top to bottom: non-calibrated parameters; calibrated fiber aspect ratio; all matrix parameters and fiber aspect ratio calibrated. Material model—color lines: 0°—red lines, 45°—green lines, 90°—blue lines. Experimental data—black dashed lines.

Identification of the model parameters was performed to find a material model suitable for describing the mechanical properties of short-carbon fiber reinforced PA6 with different percentages of fiber mass fraction (Figure 16). The stress–strain curves corresponding to calibrated material models based on experimentally obtained fiber and matrix properties are presented in the first row of Figure 16. The curves overestimated stiffness values for the materials, especially for samples at 0°. The most important influence on composite parameters was fiber size.

The second row of Figure 16 shows the material models based on the mean reverse-engineered values of the AR of the fibers (Table 3). CV in Table 3, and below, is the coefficient of variation, which is the ratio of the standard deviation to the mean value of the corresponding quantities.

The mean AR value for all reinforced PA6 material models was approximately 15, which constitutes 57% of the 26.5 value, equivalent to the experimentally observed distribution (Figure 10). Hence, the calculated effective fiber length (1) in this study was 0.57.

The third row shows the models constructed with mean value parameters after approximation of all matrix elastoplasticity model parameters and fiber AR (Table 4). The particularity in the construction of Figure 16 is that the average values of the parameters were taken from every material model to build a mean model that describes the mechanical characteristics of the reinforced PA6 with a range of fiber mass fractions from 10 to 30%.

The mean relative error of tension stress–strain curve fitting, using the mean models obtained, is presented in Table 5. The power, exponential, exponential and linear hardening laws had similar mean relative error values. In this study, the shape of the matrix stress–strain curve was more consistent with the exponential law. Adding a linear function to the exponential hardening law increased the stress–strain curve approximation accuracy only if the model parameters were calibrated in the composite tension experiment. The exponential and linear hardening law model, based on experimentally obtained matrix parameters, increased the composite curve approximation error due to the low precision in describing the slope at the end of the matrix stress–strain curve.

**Figure 17:**
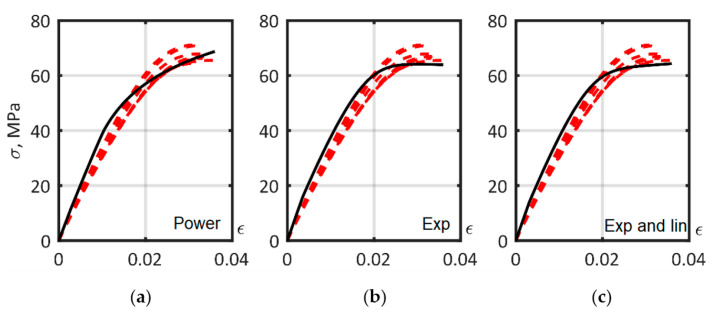
Matrix models (solid lines) after the composite stiffness calibration with different hardening laws: (**a**) power; (**b**) exponential; (**c**) exponential and linear. Dashed lines—experiment.

Figure 17 shows the PA6 matrix stress–strain refined curves after curve fitting of the composites according to the mean parameters model (Table 4). It was found that, for a better description of the parameters of the composite material, the stiffness of the matrix was overestimated during the calibration process.

### 3.8. Failure Criterion Parameter Identification for Short-Fiber Reinforced Thermoplastic Composites

For strength prediction, the Tsai–Hill 3D transversely isotropic strain-based failure criterion was applied to the reinforced PA6 (composite level) in the local finite element coordinate system (local axes) [38]. The critical fraction of failed pseudo-grains was 0.75, and multilayer failure occurred using the all-layer failure condition. The results of the identification of the model parameters are presented in Table 6.

**Figure 18:**
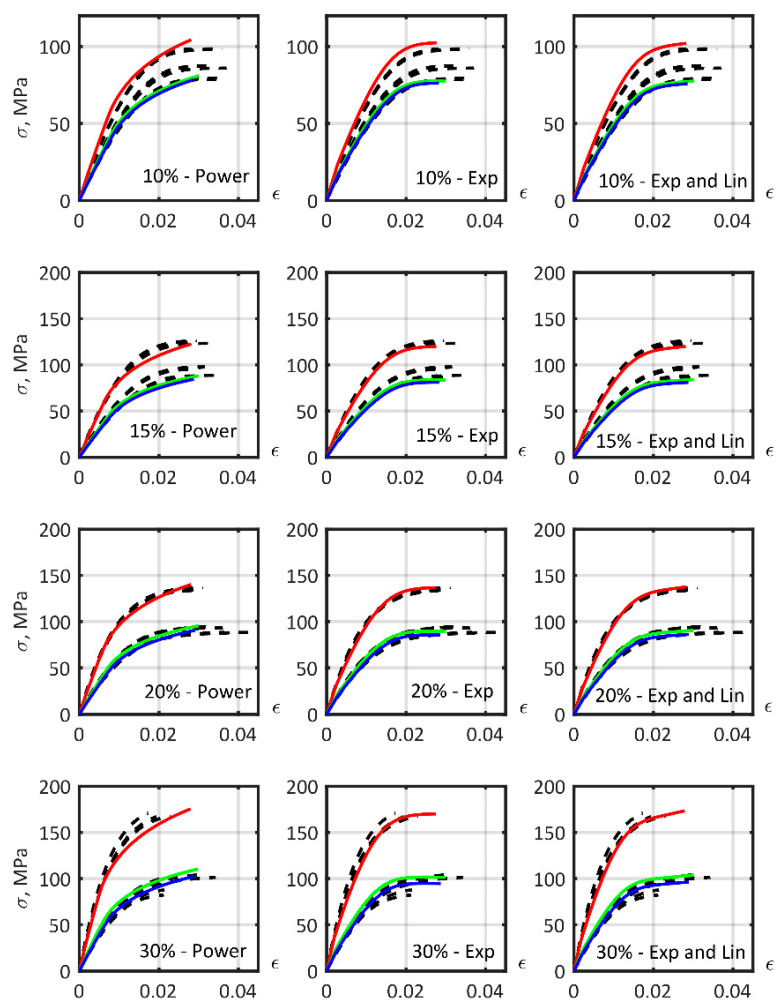
Stress–strain curves prepared from the mean obtained strain limits, and using Tsai–Hill 3D transversely isotropic strain-based values for 10%, 15%, 20%, and 30% fiber mass fractions. Material model—color lines: 0°—red lines, 45°—green lines, 90°—blue lines. Experimental data—black dashed lines.

The mean strain limits for the ultimate tensile strength of short-fiber reinforced PA6 were close to those of the different plasticity models, and constituted axial tensile 0.02, in-plane tensile 0.032, and transverse shear 0.051 (Table 6). Figure 18 shows material models with different fiber mass fraction stress–strain curves, constructed using the mean parameters of the power, exponential and exponential and lineal hardening laws exposed in Table 4, and selected failure criterion. The mean relative error of the failure criteria for fracture strain is shown in Table 7.

## 4. Discussion

A general model material for short-fiber reinforced polyamide 6 with carbon-fiber mass fractions from 10% to 30% was constructed using a second-order Mori–Tanaka homogenization scheme. The matrix was modeled using the J2 plasticity model along with power, exponential, and exponential and linear hardening laws. The mechanical characteristics of the matrix, the fiber aspect ratio, and stress–strain curves of the composites were obtained experimentally. From the examination of the three different hardening laws, it was found that the exponential law described the PA6 stress–strain curve with considerable accuracy. Furthermore, the addition of a linear term into the hardening law (exponential and lineal) increased the accuracy of model parameters that were previously reverse-engineered.

**Figure 19:**
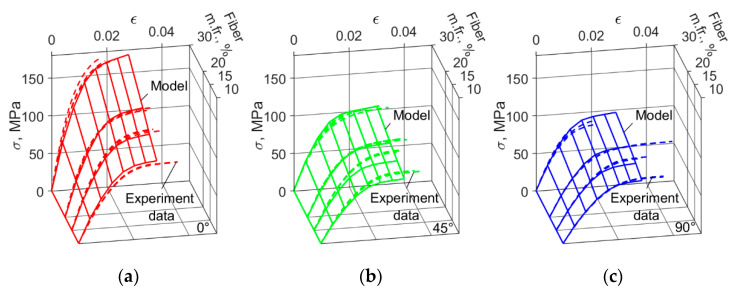
Common mass fraction composite material models for all fibers. Exponent and linear hardening law case. For angles between the flow and load directions: (**a**) 0°; (**b**) 45°; (**c**) 90°.

The results from general calibration produced models for PA6 reinforced composite which could determine the mechanical characteristics of materials with different fiber mass fractions. The novelty of this approach lies in the transition from two-dimensional (stress–strain) to three-dimensional (stress–strain–fiber fraction) approximation space (Figure 19). 

It was found that the use of the experimental AR value in the construction of the models increased the strength and stiffness of 0° samples by a factor of 1.7. Such a deviation may have been caused by a failure to account for fibers with a low AR during the electron microscopy analysis; however, a more probable reason is the inadequate consideration of the influence of including the closed surface (ellipsoid) [32] and its ideal solution in the Mori–Tanaka homogenization scheme. Moreover, the introduction of an equivalent AR value was able to replace the AR distribution, simplifying the material model. The relative effective fiber length, i.e., the ratio between the equivalent and experimental ARs, was φ = 0.57, which corresponds to a virtual length reduction of the fiber, at both ends, of approximately 21.5%.

The mean values for the material models obtained after performing curve fitting to the reinforced matrix’s parameters were used for the construction of the mean models. A mean model was produced that was capable of modeling reinforced PA6 across a wide range of fiber mass fraction, from 10 to 30%, and allowed the prediction of the mechanical characteristics with a mean relative error of 6.60%.

The calibration of mean Tsai–Hill strength failure criteria based on strain, via a transversely isotropic statement, was performed, and produced a description of the strength of reinforced PA6 with 10 to 30% carbon fiber mass fractions. The calibrated strength failure criteria could be used only as a first approximation for the failure of carbon-reinforced PA6 structures because the fracture strain mean relative error value, the large dispersion in the experimental fracture strain values, and the question of failure criteria require further investigation.

## 5. Conclusions

The Mori–Tanaka model allowed us to accurately predict the stiffness of a short-reinforced composite with an arbitrary fraction of reinforcing fibers, but required curve fitting for the whole composite because using fiber and matrix parameters obtained experimentally led to an erroneous overestimation of the composite’s mechanical characteristics. Fiber elongation had a decisive influence on the stiffness of the composite. It was found that for the correct prediction of the mechanical characteristics of a composite using the Mori–Tanaka model, it was necessary to significantly reduce the fiber aspect ratio. The exponential hardening law of the matrix provided a good description of the tensile diagram of the composite. Adding a linear term and switching to an exponential and linear model allowed an even more accurate demonstration of material behavior when approaching the strength limit, but required attention to the calibration of the slope of the tensile curve at high strain values.

When dealing with different fiber fractions, adding a third dimension to the two-dimensional stress–strain space in the form of a fiber fraction allowed us to combine the flexibility of the Mori–Tanaka model with the accuracy of calibrated tension curves from a single fiber fraction composite material. This makes it possible to improve the accuracy of the selection of the fiber fraction in material to be used in the construction of structures during the early stages of design.

In this work, averaging of the parameters of the tension curves calibrated for individual volume fractions was used to construct a three-dimensional, calibrated, Mori-Tanaka model. In the future, we plan to develop an algorithm and software that would allow simultaneous calibration of three-dimensional models over the entire volume of experimental data available.

## Figures and Tables

**Table 1:** Calculated fiber orientation tensor components.

Layer	Thickness, mm	a_11_	a_22_	a_33_	a_12_	a_13_	a_23_
1	0.1667	0.603	0.304	0.0929	0.00104	0.00019	0.00011
2	1.5003	0.821	0.126	0.0533	0.00087	0.00001	0.00003
3	0.1667	0.711	0.244	0.0451	−0.00131	−0.00033	0.00008
4	0.3334	0.461	0.486	0.0526	−0.01870	−0.00113	−0.00495
5	0.1667	0.711	0.244	0.0451	−0.00131	−0.00033	0.00008
6	1.5003	0.821	0.126	0.0533	0.00087	0.00001	0.00003
7	0.1667	0.603	0.304	0.0929	0.00104	0.00019	0.00011

**Table 2:** Calibrated pure PA6 tension curve parameters of elastoplastic matrix material models.

Hardening Law	Power	Exponential	Exponential and Linear
Young’s modulus, MPa	3341	3547	3552
Yield stress, MPa	12.57	8.27	6.68
Hardening modulus, $R_{\infty }$ MPa	-	60.59	55.41
Hardening Exponent m	0.21891	433.69	528.67
Linear hardening modulus k, MPa	158.12	-	623.17
Relative error, %	5.32	4.81	4.77

**Table 3:** Fiber aspect ratio (AR) calibration for composites with different fiber mass fraction.

Hardening Law	Fiber Mass Fraction, %				Mean AR Model	Param. CV, %
	10	15	20	30		
	Aspect ratio					
Power	14.00	16.53	14.47	14.82	15.00	7.35
Exponential	14.53	16.94	15.14	15.47	15.52	6.60
Exponential and linear	14.30	16.75	14.71	15.27	15.30	7.03

**Table 4:** Calibration of all parameters of the matrix elastoplasticity model and fiber aspect ratio.

Parameter	Fiber Mass Fraction, %				Mean Parameters Model	Parameters CV, %
	10	15	20	30		
Power Law						
Young’s modulus, MPa	4383	4437	4162	3759	4185	7.4
Yield stress, MPa	12.3	12.6	10.2	9.9	11.2	12.3
Hardening modulus, MPa	147.6	161.9	124.6	137.0	142.8	11.1
Hardening exponent	0.1995	0.2238	0.2023	0.2475	0.2183	10.2
Fibers’ AR	8.84	12.23	14.19	16.79	13.01	25.8
Exponential Law						
Young’s modulus, MPa	4654	4615	4741	3994	4501	7.6
Yield stress, MPa	13.8	21.2	12.4	16.1	15.9	24.3
Hardening modulus, MPa	59.2	51.0	51.5	38.7	50.1	17.0
Hardening exponent	404.8	370.6	353.6	377.8	376.7	5.7
Fibers’ AR	9.02	12.25	13.37	16.62	12.8	24.4
Exponential and linear law						
Young’s modulus, MPa	4672	4842	4625	3994	4533	8.2
Yield stress, MPa	13.6	13.0	14.5	14.5	13.9	5.4
Hardening modulus, MPa	56.6	54.9	46.1	37.0	48.6	18.6
Hardening exponent	447.1	417.7	381.1	458.3	426.0	8.1
Hardening linear modulus, MPa	208.8	216.0	144.1	188.4	189.3	17.1
Fibers’ AR	8.82	12.36	13.80	16.54	12.9	24.9

**Table 5:** Mean relative error of stress–strain curve prediction of different fiber mass fraction composites with models based on fibers and matrix characteristics obtained experimentally, mean fiber aspect ratio calibrated models, and the mean parameter model.

Hardening Law	Fiber Mass Fraction, %				Mean Level of Error, %
	10	15	20	30	
Models, based on fibers and matrix characteristics obtained experimentally					
Power	29.0	25.7	35.8	35.9	31.6
Exponential	27.8	25.2	33.7	34.7	30.4
Exponential and linear	28.6	25.9	36.1	35.7	31.6
Mean fiber aspect ratio calibrated models					
Power	11.7	12.1	12.0	11.1	11.7
Exponential	11.5	12.3	11.3	11.7	11.7
Exponential and linear	11.7	12.5	12.6	12.2	12.3
Mean with the calibration of all matrix and fiber aspect ratio parameters					
Power	7.4	7.6	4.9	7.7	6.9
Exponential	6.9	6.8	4.1	8.0	6.5
Exponential and linear	7.0	6.9	3.9	7.9	6.4

**Table 6:** Failure criteria strain limits for different fiber mass fraction cases.

Parameters	Fiber Mass Fraction, %				Mean Strain Limits	Parameters CV, %
	10	15	20	30		
Power law						
Axial tensile strain limit, 10^−2^	2.009	2.957	1.944	1.880	2.197	23.16
Inplane tensile strain limit, 10^−2^	3.516	3.855	3.573	2.151	3.274	23.30
Transverse shear strain limit, 10^−2^	5.606	5.140	5.235	4.364	5.086	10.26
Exponential law						
Axial tensile strain limit, 10^−2^	1.566	2.353	1.832	2.031	1.945	17.05
Inplane tensile strain limit, 10^−2^	2.999	3.524	3.430	2.278	3.058	18.57
Transverse shear strain limit, 10^−2^	5.873	5.249	5.355	4.076	5.138	14.77
Exponential and linear law						
Axial tensile strain limit, 10^−2^	2.141	2.402	1.908	1.944	2.099	10.81
Inplane tensile strain limit, 10^−2^	3.684	3.573	3.559	2.012	3.207	24.90
Transverse shear strain limit, 10^−2^	5.607	5.140	5.140	4.798	5.171	6.42

**Table 7:** Mean relative error (%) of ultimate strain prediction for different fiber mass fraction composites with models based on fiber and matrix characteristics obtained experimentally, mean fiber aspect ratio calibrated models, and the mean parameter model.

Hardening Law	Fiber Mass Fraction, %				Mean Level of Error, %
	10	15	20	30	
Power	16.24	7.79	18.92	56.63	24.90
Exponential	17.30	8.87	19.73	55.04	25.24
Exponential and linear	16.26	7.79	19.02	56.36	24.86

## Data Availability

The raw data cannot be shared at this time as the data also forms part of an ongoing study.
